# Sustained dysfunctional information processing in patients with Internet gaming disorder: 6-month follow-up ERP study

**DOI:** 10.1097/MD.0000000000007995

**Published:** 2017-09-08

**Authors:** Minkyung Park, Yeon Jin Kim, Dai Jin Kim, Jung-Seok Choi

**Affiliations:** a Department of Psychiatry, SMG-SNU Boramae Medical Center; b Department of Psychiatry, Seoul St. Mary's Hospital, The Catholic University of Korea College of Medicine; c Department of Psychiatry and Behavioral Science, Seoul National University College of Medicine, Seoul, Republic of Korea.

**Keywords:** auditory oddball task, event-related potential (ERP), internet gaming disorder, P300, treatment response

## Abstract

Internet gaming disorder (IGD), defined as an inability to control Internet-based game play, leads to serious impairment in psychological and social functioning, but few studies have identified the neurophysiological characteristics of patients with IGD. The aim of this study was to determine neurophysiological markers of P300 components associated with changes in symptoms after outpatient management with pharmacotherapy in patients with IGD. The present prospective longitudinal study included 18 patients with IGD and 29 healthy controls. The patients with IGD completed a 6-month outpatient management program including selective serotonin reuptake inhibitor-based pharmacotherapy. Event-related potentials (ERPs) were acquired during the auditory oddball task. ERPs of the patients with IGD were recorded before and after treatment. Between-group differences and the pre-to-post treatment differences in P300 components were investigated using repeated-measures analysis of variance. The primary treatment outcome was a change in score on Young Internet Addiction Test between before and after treatment. At baseline assessments, the IGD group showed significantly reduced P300 amplitudes and delayed latencies at the midline centro-parietal site compared with those in the healthy controls. No significant changes in the P300 indices were observed between pre and post-treatment in the patients with IGD after 6 months of treatment, even though the patients with IGD exhibited significant improvements in their IGD symptoms. Furthermore, no significant difference in ERPs was observed between responders and nonresponders to a 6-month treatment in patients with IGD. These results suggest that reduced P300 amplitudes and delayed latencies are candidate endophenotypes in the pathophysiology of IGD.

## 1 Introduction

Internet gaming disorder (IGD) is defined as excessive, recurrent, and problematic use of Internet gaming despite its negative consequences. IGD has recently appeared in the appendix of the Diagnostic and Statistical Manual of Mental Disorders, Fifth Edition (DSM-5) for further study.^[1]^ In the past decades, neurobiological findings related to IGD have begun to accumulate; however, additional neurobiological evidence is needed to clarify the pathophysiology of IGD.

Electroencephalography (EEG) is a useful tool for assessing brain activity with high temporal resolution on the order of milliseconds. EEG is generated by the summed postsynaptic potentials in the dendrites of pyramidal cells aligned perpendicular to the cortical surface. Several studies have used quantitative EEG (QEEG) to assess patients with IGD and reported a decrease in absolute power on the beta frequency band on the resting-state QEEG of patients with IGD compared with that of healthy controls (HCs).^[2,3]^ A recent study showed differences in the resting-state QEEG of patients with IGD and alcohol use disorder (AUD), suggesting the unique characteristics of IGD as a behavioral addiction, which is distinct from AUD.^[4]^ Furthermore, event-related potentials (ERPs), which represent averaged time-locked EEG activity related to sensory, motor, or cognitive events, provide information about the neuronal activity underlying specific cognitive processes.^[5]^ Therefore, the ERP method is considered to provide an index of information processing.^[6]^ The requirement of the oddball paradigm is to discriminate infrequent “target” stimuli from more frequently occurring “nontargets.” The P300 wave is a late cognitive ERP component associated with context updating and allocation of attentional resources.^[7]^ The amplitude of the P300 component is classically enhanced when a target is detected compared with that of a nontarget.^[8]^ Some studies have reported that P300 amplitude is impaired in patients with IGD, AUD, or heroin dependence.^[9–11]^ A meta-analysis of 30 studies of male youths with alcoholic parents confirmed that smaller P300 amplitudes are observed in males with a family history of alcoholism than in controls.^[12]^ One study reported that patients with IGD have lower P300 amplitudes during the auditory oddball task than do HCs.^[10]^ The decrease in the P300 amplitude during the auditory oddball task reflects dysfunctional information processing related to attention or working memory in patients with IGD.^[10]^

Identifying biological markers associated with predicting the development of IGD or longitudinal changes in IGD can provide a better understanding of the pathophysiology underlying IGD. A smaller P300 at age 17 years predicts the development of alcohol or drug problems by age 20.^[13]^ Prior differences in consumption do not explain this observation, as the reduced P300 amplitude is not a consequence of alcohol or drug use. Furthermore, the reduction in P300 amplitude at age 17 years sustained at age 29 in patients with substance use disorder (SUD) suggests that the P300 index is a candidate endophenotype of SUD.^[14]^ A recent resting-state QEEG study showed that absolute delta activity is associated with changes in prospective addiction symptoms in patients with IGD after 6 months of outpatient management.^[15]^ However, to date, no study has investigated the ERP-related neurophysiological factors associated with prospective changes in patients with IGD. Thus, the present study aimed to determine the neurophysiological markers associated with symptom changes during the auditory oddball task in patients with IGD and to detect markers related to the treatment response after outpatient management with pharmacotherapy. Based on previous studies of patients with IGD^[10]^ and SUD,^[13,14]^ it was hypothesized that patients with IGD would exhibit decreased P300 amplitudes and that these decreased amplitudes would be sustained after 6 months of outpatient management.

## 2 Subjects and methods

### 2.1 Study participants

This prospective longitudinal study was conducted with 47 male participants (18 with IGD, mean age: 22.61 ± 5.10 years; 29 HCs, mean age: 24.66 ± 3.80 years). All patients had sought treatment at an outpatient clinic of Boramae Medical Center, Seoul, South Korea, due to excessive participation in Internet gaming, and IGD was diagnosed by an experienced psychiatrist based on the criteria of the DSM-5.^[1]^ Additionally, Young Internet Addiction Test (IAT) was used to measure the severity of IGD symptomatology.^[16]^ The IAT items are rated on a 5-point scale ranging from 1 (very rarely) to 5 (very frequently); in the present study, IAT scores were calculated as the total score for all 20 items ranging from 20 to 100. Additionally, the Structured Clinical Interview for DSM-IV disorders was used to assess lifetime psychiatric diagnoses.

After completion of the clinical assessments and a baseline EEG scan, the patients with IGD completed a 6-month outpatient management program that included pharmacotherapy with a selective serotonin reuptake inhibitor using the following average daily doses: escitalopram at 15.83 ± 9.17 mg, fluoxetine at 50.00 ± 9.17 mg, or paroxetine at 30.00 ± 14.14 mg. All patients with IGD who completed the 6-month treatment program had comorbid depressive or anxiety symptoms at baseline and completed a follow-up EEG scan upon finishing. The primary treatment outcome of the present study was change in the IAT score from pre to post-treatment, and the Beck Depression Inventory (BDI)^[17]^ and Beck Anxiety Inventory (BAI)^[18]^ were administered at the pre and post-treatment assessments of the patients with IGD to evaluate changes in comorbid depressive and anxiety symptoms. HCs were directly recruited from the local community, did not have a history of a psychiatric disorder, and played Internet games <2 hours per day.

Participants with no history of significant head injury, seizure disorder, intellectual disability, psychotic disorder, or SUD (other than one involving nicotine) were included in this study. Additionally, all participants were medication-naïve at the time of the baseline assessment. The Korean version of the Wechsler Adult Intelligence Scale-III (WAIS-III) was administered to all participants to estimate their intelligence quotient (IQ); individuals with WAIS-III scores <80 were excluded from the present study. The investigation was carried out in accordance with the latest version of the Declaration of Helsinki. This study protocol was approved by Institutional Review Board of Boramae Medical Center. Informed consent was obtained from all participants before participation.

### 2.2 Experimental procedure

A detailed description of the experimental procedure was presented in our previous report.^[10]^ A pseudorandom sequence of deviant stimuli (15%) and standard stimuli (85%) were presented binaurally by a STIM 2 sound generator (Compumedics, El Paso, TX) for the auditory oddball task. In all, 300 stimuli were presented binaurally through earphones; the deviant stimulus was classified as a high-frequency tone of 2000 Hz, and the standard stimulus was classified as a low-frequency tone of 1000 Hz. Each stimulus had a duration of 100 ms (10-ms rise and fall times) with fixed intertrial intervals of 1250 ms. The participants were instructed to press a response-box button with their right hand in reaction only to the high-pitched sound. The speed and accuracy of the response were emphasized.

### 2.3 EEG recording

The ERPs were recorded from patients with IGD before and after the 6-month treatment. The EEG was acquired using a 64-channel Quick-cap system (Compumedics), which refers to the linked mastoid in an isolated sound-shielded room with the ground channel located in an electrode cap between FPz and Fz. Horizontal and vertical electro-oculograms (EOGs) were recorded from electrodes positioned at the outer canthus of each eye, and above and underneath the left eye. The sampling rate for EEG recording was 250 or 500 Hz. All data were processed with a 0.3 to 100 Hz bandpass filter. The impedance of all electrodes was <10 kΩ.

### 2.4 ERP analysis

The EEG signals were further processed off-line using Curry 7 software (Compumedics). Recordings were first down-sampled to 250 Hz. Data were then re-referenced to a common average reference and filtered using a bandpass frequency of 0.3 to 30 Hz. All EEG and EOG recordings were visually inspected to reject gross artifacts, such as those involving movement. Eye blinks and eye movements were corrected based on the artifact reduction method developed by Semlitsch et al.^[19]^ Data were segmented into 1000-ms epochs, which included the 100 ms before stimulus onset. All segments with voltage ≥70 μV were considered artifacts and automatically discarded from further processing. Trials with response times >800 ms were considered error responses and were rejected. Only those trials with correct responses to the infrequent stimuli were averaged and analyzed. A region of interest including 2 midline centro-parietal electrodes of maximal voltage was chosen (CPz and Pz). The ERP waveforms of each participant had a minimum of 30 artifact-free trials. The auditory P300 was identified as the most positive peak in a 248 to 500-ms time window after stimulus onset. Topographic maps were created using Matlab 7.10.0 (MathWorks, Natick, MA) and the EEGLAB toolbox.^[20]^

### 2.5 Statistical analysis

One-way analysis of variance (ANOVA) was used to compare group differences in the demographic and clinical data. One-way analysis of covariance (ANCOVA) was used, with age and IQ as covariates, to compare group differences in the behavioral data. A repeated-measures ANCOVA was performed for the P300 amplitudes and latencies with electrode site as a within-subject factor, group as the between-subject factor, and age, IQ, BDI, and BAI as covariates. We evaluated the effects of treatment separately for the P300 amplitudes and latencies using repeated-measures ANCOVA with electrode site as the within-subject factor and treatment (pre vs post-treatment) as the between-subject factor. The relationship between ERP changes and addiction symptom changes after treatment was analyzed by Pearson correlation analysis. A *P* value <.05 was considered significant. All statistical analyses were performed using SPSS v20.0 software (SPSS Inc., Chicago, IL).

## 3 Results

### 3.1 Demographic and clinical data

The demographic and clinical characteristics of the participants are presented in Table 1. No significant differences in age or IQ were observed between the IGD and HC groups, but the IGD group had a lower education level (*P* < .001) than the HC group. The IGD group had higher BDI (*P* < .001) and BAI (*P* < .001) scores than the HC group. No significant differences in BDI or BAI scores were observed between the pretreatment IGD and post-treatment IGD groups. The primary treatment outcome was a change in score on the Young IAT from before and after treatment (*P* = .004).

**Table 1:**
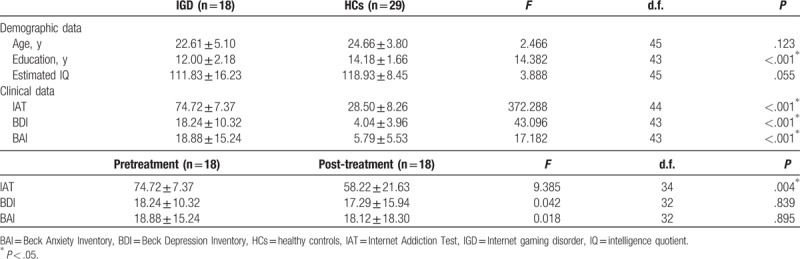
Demographic and clinical characteristics of study subjects.

### 3.2 Behavioral measures before and after treatment

The behavioral performance data are presented in Table 2. No significant group (HCs vs pretreatment IGD, pretreatment IGD vs post-treatment IGD) differences with regard to accuracy rate and reaction times were observed.

**Table 2:**
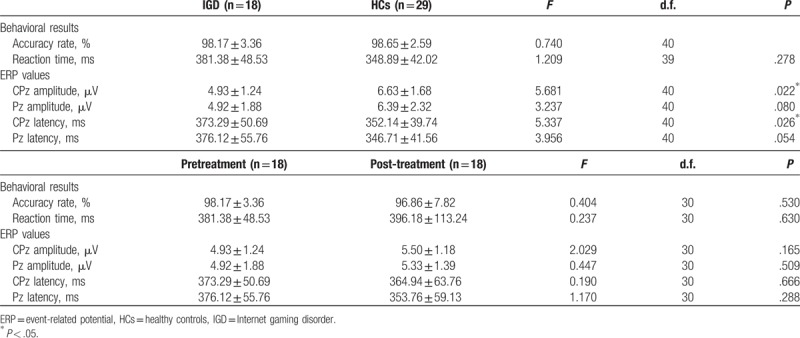
Behavioral results and P300 values.

### 3.3 ERP values in the 2 groups at the baseline assessment

**Figure 1:**
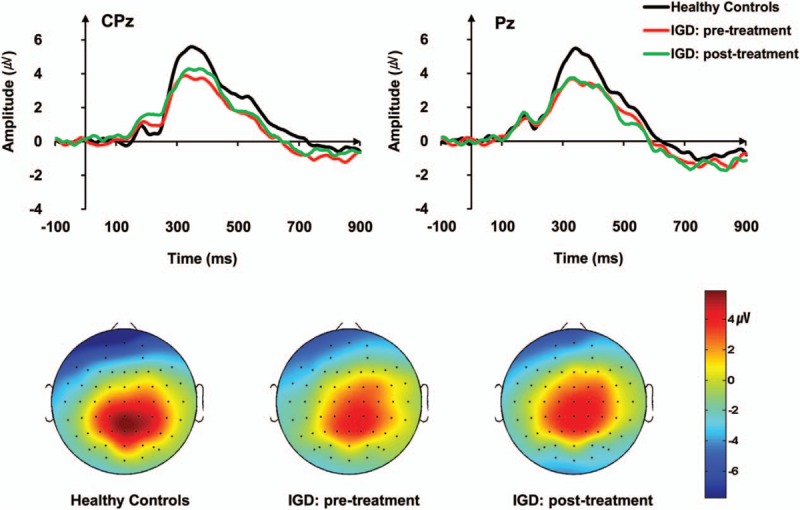
Grand-average event-related potential (ERP) waveforms to deviant tones in the auditory oddball task for patients with pre and post-treatment Internet gaming disorder (IGD) and healthy controls (HCs).

The grand-average ERP waveforms for deviant stimuli are shown in Fig. 1. Significant main effects of group (*F*_[1, 40]_ = 5.458, *P* = .025_ for P300 amplitudes were found. There were no main effect of electrode site and no interaction with the P300 amplitudes. Post hoc tests revealed that the IGD group showed significantly lower P300 amplitudes than those of the HCs at CPz (*F*_[1, 40]_ = 5.681, *P* = .022), but not at Pz. Significant main effects of group (*F*_[1, 40]_ = 5.269, *P* = .027) for P300 latencies were found. There were no main effects of electrode site and no interaction with the P300 latencies. Post hoc tests revealed that the IGD group had significantly longer P300 latencies than those of the HCs at CPz (*F*_[1, 40]_ = 5.337, *P* = .026], but not at Pz.

### 3.4 ERP values before and after treatment in the IGD group

None of the main effects or interactions was statistically significant for the P300 amplitudes or P300 latencies. No significant changes in P300 amplitudes or P300 latencies were observed between before and 6 months after IGD treatment, even though the patients with IGD exhibited significant improvements in their IGD symptoms as measured by Young IAT. In further analysis, no significant differences in ERP were observed at baseline between responders (decrease of IAT scores ≤30% relative to baseline IAT scores, n = 10) and nonresponders after the 6-month treatment in patients with IGD (n = 8).

### 3.5 Relationship between ERP changes and addiction symptom changes after treatment

No significant correlations were observed between P300 amplitudes or latencies at the CPz site or IAT scores at baseline in patients with IGD. Changes in P300 amplitudes and latencies after treatment in the IGD were not correlated with changes in the IAT score.

## 4 Discussion

The present study found that decreased P300 amplitudes and delayed latencies in patients with IGD were sustained after treatment with pharmacotherapy, even though the patients’ IGD symptoms improved significantly after treatment. This finding indicates that the decreased P300 amplitudes and delayed latencies at the baseline assessment, and sustained dysfunction after treatment in patients with IGD are reliable neurobiological markers for IGD and are associated with vulnerability to develop the disorder. This is the first study to investigate prospective ERP changes associated with symptomatic improvements in patients with IGD.

Identifying biological markers associated with treatment-related changes can provide a better understanding of the pathophysiology underlying IGD. Because IGD is associated with a poor quality of life and lower psychological well-being,^[21]^ identifying vulnerable individuals early and implementing early interventions involving individualized treatment approaches would likely have significant clinical importance. Of the various biological markers, those related to vulnerability to develop the disorder are considered the endophenotype. The endophenotype is stable over time, and those who possess the endophenotype, state independent whether illness is present or in remission phenotypically, are at high risk of developing psychiatric disorders.^[22]^ Furthermore, a reduction in P300 amplitude in childhood could increase the odds of SUD in adolescence or late adolescence.^[23–25]^

In the present study, we found decreased P300 amplitudes and delayed latencies at the baseline assessments and sustained dysfunction after treatment in patients with IGD during the auditory oddball task. Abnormal changes in the P300 measurements during the auditory oddball task reflect that patients with IGD demonstrate cognitive deficiencies in the capacity and speed of information processing. These results are consistent with a previous ERP study.^[10]^ In addition, the neurotransmitter mechanisms underlying the generation of P300 are as yet unclear, but several researches suggest some neurotransmitter-mediated P300 generations have been implicated. Striatal dopamine D2/D3 receptor status is positively correlated with P300 amplitude and negatively correlated with P300 latency in patients with depression, and those evidence indicates that dopaminergic activity plays a role in the generation of the P300.^[26]^ Some studies have also found evidence that IGD is associated with deficit in the dopaminergic neural system. In a previous positron emission tomography (PET) imaging study, reductions in dopamine D2 receptors availability in subdivisions of the striatum, including the bilateral dorsal caudate and right putamen, have been observed in patients with IGD.^[27]^ Patients with SUD were also found to have P300 dysfunctions associated with the dopaminergic brain systems.^[28]^ Furthermore, in this study, we found no correlations between ERP changes and addiction symptoms in patients with IGD and no differences in ERP measures between treatment responders and treatment nonresponders in patients with IGD. Wan et al^[29]^ reported significantly reduced P300 amplitudes at Pz in patients with SUD who did not complete treatment compared with those who did. Those authors also suggested that P300 amplitude is a good candidate for predicting treatment completion, as reduced P300 amplitude has been consistently observed in patients with SUD.^[29]^ However, we found no differences in ERP amplitudes or latencies according to the treatment responses in patients with IGD. Taking these findings together, the neurophysiological characteristics according to genetic status of the dopaminergic system must be examined to clarify the role of ERP measures in the IGD endophenotype.

The present study had some limitations. First, the sample size was too small to represent the overall characteristics of patients with IGD, and only male subjects were included. Thus, the generalizability of the results is limited. Second, the treatment modalities were not well-organized, but consisted of typical outpatient care. However, the main focus of this study was to determine the ERP markers associated with longitudinal changes in symptoms rather than the effects of the treatment itself. In the future, it will be necessary to investigate the effects of specific treatment modalities on the neurophysiological markers of patients with IGD.

## 5 Conclusions

The main overall finding from this study is that decreased P300 amplitudes and delayed latencies were sustained in patients with IGD after a 6-month treatment including pharmacotherapy, even though their IGD symptoms significantly improved after treatment. In addition, no correlations were detected between the P300 indices and addiction symptoms, and no differences in baseline P300 indices were observed between treatment responders and treatment nonresponders in the patients with IGD. Therefore, reduced P300 amplitudes and delayed latencies could be candidate endophenotypes in the pathophysiology of IGD.
